# Vestibulocochlear system and quality of life in musicians: a study on the effects of auditory exposure

**DOI:** 10.1590/2317-1782/e20240010en

**Published:** 2025-02-03

**Authors:** Magali Scheuer, Bianca Nunes Pimentel, Priscilla dos Santos Martins, Hélinton Goulart Moreira, Valdete Alves Valentins dos Santos

**Affiliations:** 1 Programa de Pós-graduação em Distúrbios da Comunicação Humana, Universidade Federal de Santa Maria – UFSM - Santa Maria (RS), Brasil.; 2 Faculdade Unyleya - Rio de Janeiro (RJ), Brasil.; 3 Curso de Fonoaudiologia, Departamento de Fonoaudiologia, Universidade Federal de Santa Maria – UFSM - Santa Maria (RS), Brasil.

**Keywords:** Hearing, Vestibular Function Tests, Quality of Life, Music, Noise

## Abstract

**Purpose:**

To evaluate and compare the effects of sound exposure on the vestibulocochlear system and the quality of life among musicians.

**Methods:**

Fifty-six individuals participated, including 28 musicians and 28 non-musicians, aged 18 to 45 years, of both genders. Participants underwent medical history assessment, basic audiological evaluation, vestibular evoked myogenic potential (VEMP), and exclusively, WHOQOL-Bref questionnaire.

**Results:**

Professional and enthusiast musicians participated, showing increased auditory thresholds at 3 and 4 kHz frequencies and considerably satisfactory quality of life. There was significance in cervical VEMP latencies and the latency of the P15 wave in the left ocular VEMP of the study group compared to the control group. VEMP demonstrated larger waves bilaterally in men compared to women.

**Conclusion:**

It was concluded that sound exposure, within tolerance limits in terms of time and intensity, was capable of providing auditory, vestibular, and quality of life benefits for musicians.

## INTRODUCTION

Music and human beings are closely linked, that is, music has always been present in the history of humanity. Currently, music is a professional occupation for millions of people around the world, acting in various areas and representing an important sociocultural role. For this reason, when these subjects are exposed, several benefits can be observed, mainly related to mechanisms that facilitate emotional expression and regulation, the strengthening of social ties, the feeling of belonging, as well as the promotion of creativity and cognitive development^(1)^.

It is known that music has beneficial effects on quality of life (QoL) and that musical practice sensitizes auditory perception through the perceptive and physiological selectivity of sounds at the cochlear level. This promotes cortico-cochlear activation during musical training, proportional to the time of exposure to practice^(2)^. In this way, auditory neuroplasticity acts to improve cochlear adjustment and increase spectral acuity, improving auditory perception.

On the other hand, musicians are classified as a risk group for the development of occupational disorders. If such professionals are exposed inappropriately and for long periods to high levels of sound pressure, such exposure can trigger auditory and extra-auditory damage (headache, alterations in the immune system, insomnia, among others). Possible musculoskeletal problems also stand out when related to inadequate posture and effort, making it interesting to measure the vestibulocochlear system and QoL in this population^(3,4)^.

For musicians, knowledge of the effects of musical exposure on auditory function is essential, but it is still incipient with regard to vestibular health (postural balance). Recent studies show that inadequate sound exposure induces hearing alterations and also affects the vestibular organs (saccule and utricle)^(5,6)^. In this sense, vestibular symptoms may precede hearing loss, that is, possible deficits related to hearing health caused by professional practice, in addition to musculoskeletal and emotional problems, together, may interfere with the professional career and quality of life, given the importance of auditory and body perception for this professional category.

Considering the close anatomical and physiological relationship between the auditory and vestibular systems, inadequate musical exposure can affect both the cochlea and the saccule and utricle, which are primarily responsible for obtaining linear acceleration variations (horizontal and vertical) of the head and body^(3)^. In view of this, the possibility of using the Vestibular Evoked Myogenic Potential (VEMP) is highlighted, which provides an understanding of body balance reflexes (vestibulo-ocular reflex and vestibulo-cervical reflex), which act directly on body balance.

Since research on auditory and vestibular function of musicians is still incipient when measured and related to quality of life, the present study aimed to evaluate and compare the effects of sound exposure on the vestibulocochlear system between groups and the quality of life of musicians.

## METHOD

This study was approved by the Research Ethics Committee, under opinion number 2,732,475 and CAAE 87348618.3.0000.5346. This is a cross-sectional, observational, descriptive study, which used a quantitative method, developed in the otoneurology outpatient clinic of the institution of origin, in the pre-pandemic period.

The sample was composed by convenience, and all participants performed the procedures individually, on a previously scheduled day and time. Furthermore, all participants consented to the research by reading and signing the Free and Informed Consent Form – FICF. Non-musicians were selected on a voluntary basis.

The following inclusion criteria were adopted:

Control group (CG): Ages between 18 and 45 years; hearing thresholds within normal standards; tympanometric curves indicating normal mobility of the tympanic-ossicular system; presence of normal levels of contralateral acoustic reflexes; presenting mental and cognitive conditions to respond and perform the reproduction of the requested commands; not presenting complaints or history of neurological, traumatic, cervical or ocular alterations that would make it impossible to perform the proposed procedures, as well as chronic diseases; not having exposure to physical, chemical and biological risks, in accordance with the tolerance limits set forth in Regulatory Standard 15^(7)^; avoiding the use of vestibulotoxic and relaxing drugs for 48 hours before the exams;
Study group (SG): In addition to the criteria common to the groups (the same listed for the CG), the following was included for this group, given the research objective of highlighting audiological findings in musicians: individuals could present hearing thresholds equal to or greater than 30 dBHL at any hearing frequency assessed by conventional pure tone audiometry (250 to 8000 Hz), presence of auditory and vestibular complaints after starting musical practice, and must be a music professional (musician), and have worked for more than six months^(8)^.

A total of 59 individuals were assessed, of which 56 met the inclusion criteria and were divided into two groups, matched for sex and age:

Study Group (SG): 28 musicians participated, 21 (75%) male and seven (25%) female, with an average age of 25.07 years;
Control Group (CG): Composed of 28 individuals, who were not music professionals, or who did not play any type of musical instrument.

All subjects underwent the following procedures:

**Previously structured anamnesis:** it was prepared for the occasion with questions relating to clinical history, hearing and occupational health, past and present. Furthermore, musicians were asked about aspects related to musical practice;
**Inspection of the external auditory canal:** performed with the MD 2.5V Omni 3000 LED Fiber Optic otoscope, in order to check for possible obstructions that would make it impossible to perform the examinations;
**Pure Tone Audiometry:** performed using an Interacoustics AD629 audiometer with TDH-39P shell headphones. Hearing frequencies from 250 to 8000Hz were assessed via air conduction and, when necessary, bone conduction research was performed, the results of which were interpreted according to the standards for hearing loss, regarding type and degree^(9)^;
**Speech audiometry:** the speech recognition threshold was investigated using a list of disyllabic words presented live, considering as a result the intensities (dB) at which individuals achieved 50% correct answers to the words presented. To assess the percentage rate of speech recognition, the repetition of a list of 25 monosyllabic words presented aloud was requested, considering the results with no difficulty in understanding speech as those who presented 100% to 92% correct answers^(9)^;
**Acoustic immittance measurements** (tympanometry and contralateral acoustic reflexes): were performed using the AT235 equipment, from Interacoustics, and TDH-39 type headphones. For the classification of tympanometric curves and acoustic reflexes, the criteria already proposed in the literature were used^(9)^;
**Vestibular evoked myogenic potentials:** Cervical and Ocular Vestibular Evoked Myogenic Potentials were performed using the MASB ATC Plus equipment, from Contronic. First, the participant's skin was cleaned with absolute alcohol, followed by abrasive paste and fixing of the electrodes with electrolytic paste. It is noteworthy that to perform the two vestibular myogenic potentials, the individuals were seated and that all participants, from both groups, received instructions regarding the intensity of presentation of the VEMP sound stimuli. No verbal or non-verbal expression of auditory discomfort was presented during the assessment.

Both potentials were performed with tone burst auditory stimulus at an intensity of 118 dBHL and a frequency of 500 Hz, presenting a total of 200 stimuli, with a presentation speed of 5.1 stimuli per second, with a band-pass filter from 10 to 1,500 Hz. The electrode impedance test was less than 5 KΩ, and the difference between them was less than 2 KΩ^(10)^.

Two stimulations were presented on each side, in order to verify the replicability of the responses, with intervals between them, allowing the muscles to rest in between.

**Cervical Vestibular Evoked Myogenic Potential (cVEMP):** The ipsilateral active electrode was positioned on the anterior border of the sternocleidomastoid muscle, in its middle third. The ipsilateral positive reference electrode was positioned above the upper edge of the clavicle, on the sternal line, and the ground electrode was fixed to the forehead (Fpz). Participants were instructed to turn their head horizontally to the opposite side to the stimulated ear during sound stimulation, contracting the sternocleidomastoid muscle. The latencies in milliseconds (ms) of the first positive peak and the first negative valley, called P13 and N23^(10,11)^, were marked.
**Ocular Vestibular Evoked Myogenic Potential (oVEMP):** The ipsilateral active (negative) electrode was placed approximately 1 cm below the lower eyelid, on the inferior oblique muscle. The contralateral reference electrode (positive) was placed at a distance of 1 cm from the active electrode, and the ground electrode was fixed to the forehead (Fpz). Participants were instructed to keep their heads upright and only look upwards until the end of the sound stimulation. The latencies in milliseconds (ms) of the first negative valley and the first positive peak, called N10 and P15, were marked bilaterally^(10,11)^.

For both cVEMP and oVEMP, the latency, peak and interpeak amplitude (interamplitude) were recorded in “microvolts” (uV). The quantitative data (latencies, amplitudes and interamplitudes) of the musicians were analyzed with the normality values ​​of the equipment of the aforementioned Laboratory and were also compared with the results of the control group. The analyses and appropriate markings on all tracings were submitted to the evaluation of two speech-language pathologist judges, both specialists in the evaluation of patients with the VEMP protocol.

For the SG, exceptionally, the following questionnaire was also applied, considering the objective of the study to measure the quality of life of only musicians after comparing the vestibulocochlear findings between musicians and non-musicians.

**Quality of Life Assessment Instrument - The World Health Organization Quality of Life (WHOQOL - Bref):** This is an abbreviated version of the World Health Organization's Quality of Life Assessment Instrument^(12)^. This instrument consists of 26 questions divided into four domains: physical, psychological, social and environmental, as well as two general questions about overall QoL. Participants responded on a scale of zero to five (the higher the score, the better the QoL), considering the last two weeks lived. The results were added according to each domain, and the final scores demonstrated the QoL of the individual evaluated. As there is no cut-off point, the closer to 100, the better the QoL.

### Statistical analysis of data

The qualitative variables relating to the sample characterization data were analyzed using descriptive statistics. To compare VEMP values ​​between groups, the Shapiro-Wilk test was first performed. This method was used to test the distribution of quantitative variables, from which the following were selected: the Mann-Whitney U test, for comparisons between two independent samples; and the Kruskal-Wallis test for multiple comparisons, using the STATISTICA 9.1 computer application. The significance level considered was 5% (α=0.05).

## RESULTS

### Sample characterization for SG

For this group, an average of 13.14 years of musical experience was obtained, with 14.96 hours of weekly sound exposure (between rehearsals and performances) distributed over an average of 5.39 days a week. In relation to the sound intensity to which they are exposed according to their own perception, there was a prevalence of high levels (57.14%) and intensity of extra-musical noise at the time of performance and a predominantly low intensity at the rehearsal (32.14%). It is worth noting that among the instrument categories, five musicians use metal, five percussion, two wood and 16 strings.

Regarding musical aspects and preferences, it was observed that a large percentage of participants work professionally, with a long period of experience and high self-perception in relation to sound exposure, distributed on average over five days a week (Table 1).

**Table 1:** Characterization of musicians according to musical aspects and preferences (n=28)

**Variable**	**Category**	**Mean (%)**
Professional instrumentalist	Yes	60.71
	No	39.29
Musical genres	Popular	46.43
	Classic	14.29
	Both	39.29
Intensity (Loudness) of sound exposure	High	57.14
	Average	32.14
	Low	10.72
Intensity (Loudness) of extra-musical noise	High	21.43
	Average	28.57
	Low	32.14
	Non-existent	17.86
Preference when playing music	Accompanied	82.14
	Solo	17.86
Position in which plays	Sitting	42.86
	Standing	17.86
	Both	39.29
Environment in which plays	Outdoors	0.00
	Indoors	100.00
	Large room	42.86
	Small room	57.14

Caption: n = sample number; % = percentage

**Figure 1:**
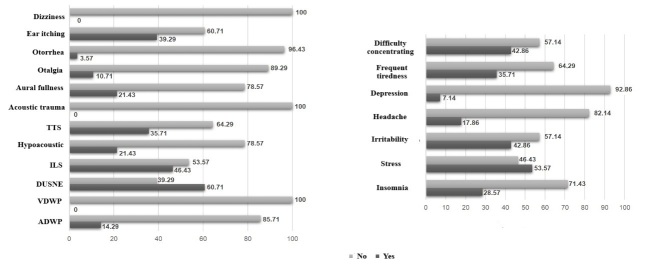
Characterization of musicians according to auditory and extra-auditory symptoms after sound exposure (n=28)

Regarding the self-perception of auditory and extra-auditory symptoms after sound exposure for the SG, it was shown that, on average, 50% of the sample perceived them. In this sample, the following were observed to have the highest incidence: difficulty understanding speech in noisy environments, intolerance to sounds, stress, irritability and reduced concentration (Figure 1).

Caption: ADWP = auditory discomfort when playing; VDWP = vestibular discomfort when playing; DUSNE = difficulty understanding speech in noisy environments; ILS = intolerance to loud sounds; TTS = temporary threshold shift; n = sample number

### Characterization of peripheral auditory acuity for the SG

For peripheral auditory acuity, in the SG, the average air-tone thresholds were within normal standards, with symmetry between the ears. However, 10.71% of musicians had a tonal threshold equal to or greater than 30 dBHL at specific frequencies of 3 and 4 kHz.

### Comparison of cVEMP results between groups

Regarding the cervical vestibular myogenic responses, statistically significant differences were evidenced for both ears, related to the latency of the P13 and N23 components, with shorter response times for the musicians (Table 2).

**Table 2:** Comparison between the results obtained in the cervical vestibular evoked myogenic potential (cVEMP), by ear, of the study and control groups (n = 56)

**Side**	**cVEMP**		**Study (n= 28)**		**Control (n= 28)**		**Z**	**p-value**
			**Mean**	**SD**	**Mean**	**SD**		
R	**P13**	**Latency**	17.51	1.21	18.99	2.24	-2.65	<0.01*
		**Amplitude**	59.36	44.40	43.45	22.53	0.98	0.32
	**N23**	**Latency**	26.65	2.43	27.82	2.42	-1.93	0.05*
		**Amplitude**	47.89	56.20	46.15	22.72	0.15	0.88
		**Interamplitude**	118.58	86.78	88.82	43.22	0.92	0.36
L*	**P13**	**Latency**	17.89	1.65	19.14	1.99	-2.60	<0.01*
		**Amplitude**	54.68	37.47	46.44	23.48	0.54	0.59
	**N23**	**Latency**	26.57	2.78	27.97	2.13	-2.28	0.02*
		**Amplitude**	43.21	59.46	55.22	27.17	-0.77	0.44
		**Interamplitude**	114.96	77.96	101.18	49.30	0.29	0.77

Mann-Whitney U test; p < 0.05

* sample number equal to 27 individuals in both groups

Caption: P = positive wave; N = negative wave; SD = standard deviation; R = right; L = left; n = sample number; Z = confidence level; p = p-value

### Comparison of oVEMP results between groups

Regarding ocular vestibular myogenic responses, significant differences were only evident for left ear latency for the P15 component, with shorter response times for SG (Table 3).

**Table 3:** Comparison between the results obtained in the ocular vestibular evoked myogenic potential (oVEMP), by ear, of the study and control groups (n = 56)

**Side**	**oVEMP**		**Study (n= 28)**		**Control (n= 28)**		**Z**	**p**
			**Mean**	**SD**	**Mean**	**SD**		
R	**N10**	**Latency**	13.02	1.31	13.73	1.34	-1.04	0.30
		**Amplitude**	2.65	3.62	3.50	4.13	-0.55	0.58
	**P15**	**Latency**	17.73	1.67	18.65	1.29	-1.53	0.12
		**Amplitude**	3.77	3.69	3.88	4.21	0.42	0.67
		**Interamplitude**	6.97	6.72	7.38	8.24	0.34	0.74
L*	**N10**	**Latency**	13.23	1.19	13.98	1.67	-1.49	0.14
		**Amplitude**	3.15	3.52	3.12	3.12	0.24	0.81
	**P15**	**Latency**	17.71	1.36	18.34	1.65	-2.32	0.02*
		**Amplitude**	4.53	4.23	4.29	5.09	0.88	0.38
		**Interamplitude**	8.09	7.21	7.98	9.53	0.75	0.45

Mann-Whitney U test; p < 0.05

* sample number equal to 28 individuals in both groups

Caption: P = positive wave; N = negative wave; SD = standard deviation; R = right; L = left; n = sample number; Z = confidence level; p = p-value

### Graphical representation of evoked myogenic potentials

**Figure 2:**
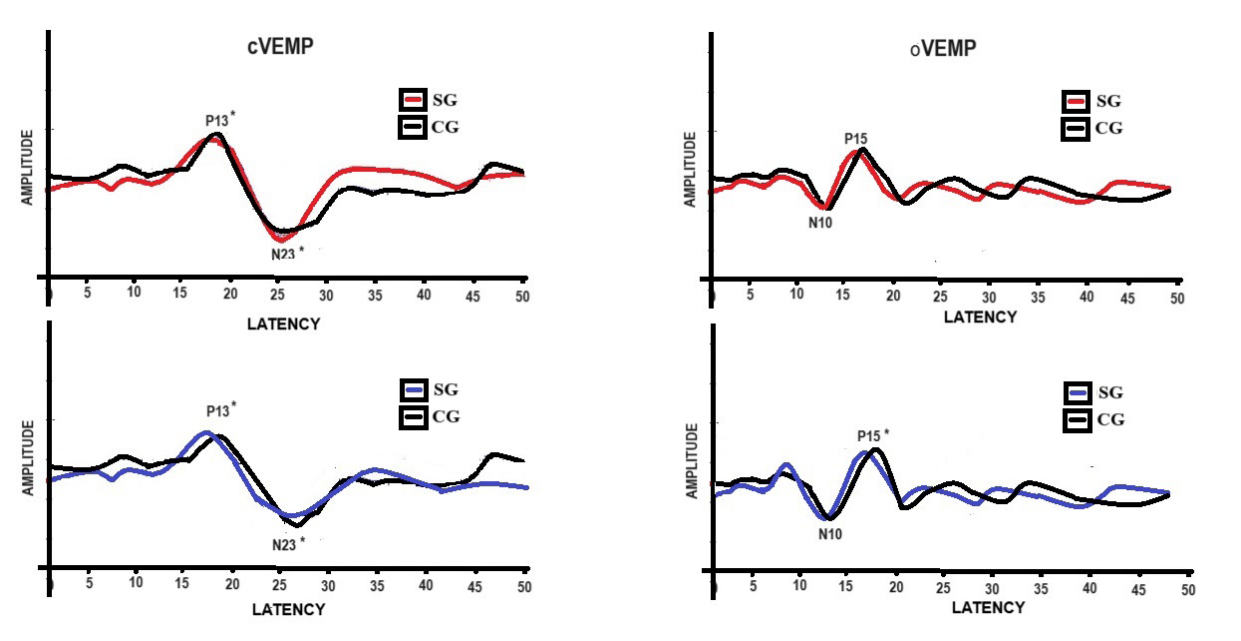
Graphical representation of cVEMP and oVEMP, by ear

Figure 2 shows the graphic representation of cVEMP, for both ears, with lower latency for SG, and the graphic representation of oVEMP, for both ears, with lower latency for P15 for SG.

Caption: cVEMP = cervical vestibular reflex; oVEMP = ocular vestibular reflex; CG = control group; SG = study group; red = right ear; blue = left ear; *statistically significant difference

### Characterization of the QoL of musicians

Regarding the QoL of musicians, the response was considerably satisfactory. According to the average scores obtained when applying the WHOQOL-Bref questionnaire, the environmental domain (61.52) presented the greatest loss, and the physical domain (75.38) the best score. Among these are the psychological domain, with 72.17, and the social domain, with 73.81, with 70.72 being the average overall QoL score.

## DISCUSSION

Musical practice is reported in specialized literature as an important instrument of neuroplastic changes in the vestibulocochlear system^(13)^. The findings indicate that correct exposure can improve working memory and stimulate motor and premotor cortical regions. As a consequence, it becomes important for the auditory and vestibular systems, due to the benefits of plasticity in these regions^(14,15)^.

Music provides changes in the sensitivity of auditory perception, through the perceptive and physiological selectivity of sounds at the cochlear level, carrying out cortico-cochlear activation and enabling greater benefits to auditory performance^(2,16)^. Therefore, auditory neuroplasticity acts to improve cochlear adjustment and increase spectral acuity, enhancing auditory perception and, consequently, contributing to a better quality of life^(17)^.

In view of such manifestations, the importance of the present research is highlighted, due to the lack of studies that seek to measure such aspects related to body balance.

### Musical exposure and auditory and extra-auditory symptoms

It was possible to observe that the musicians presented hearing complaints, mainly related to sound intolerance, vestibular discomfort and transient threshold changes after musical practice. Furthermore, extra-auditory effects related to concentration difficulties, irritability and stress were evidenced. These findings corroborate other studies that also observed the presence of these complaints^(18,19)^.

The presence of auditory symptoms becomes common in the population studied, since high levels of music and noise can permanently damage the ear and, for a time, be comfortable and even imperceptible, making protective measures difficult. One study found that a large number of American adolescents and young adults had experienced temporary threshold shifts and transient tinnitus after high levels of sound exposure^(20)^. These symptoms are justified by the hypothesis that exposure to high and continuous loudness can cause irreparable damage to some fraction of the hair cells and nerve fibers of the cochlea, even when the audiometric thresholds initially recover and the symptoms disappear^(21)^. Thus, the accumulation of damage from repeated exposures could cause more chronic hearing problems.

The emergence and possibility of chronicity of symptoms can cause several extra-auditory effects, since, despite being a leisure noise, music causes damage, even if minor ― protection of the stapedius muscle ―, but similar to other work activities^(22)^. These can be justified due to the fact that work activity is associated with stressful activities for better performance, as well as due to the impacts of noise on the auditory and bodily manifestations of these individuals, causing the self-perception of these complaints.

It is worth noting that the incidence of symptoms is proportional to the instrument used, that is, recent research shows that the symptom is directly proportional to the sound pressure produced by the instrument, as well as the material and position characteristics^(18)^. Thus, the presence of auditory and extra-auditory complaints is justified.

### Musical exposure and peripheral auditory acuity

In the present research, it was possible to observe that individuals who were enthusiastic or professional musicians presented air tonal thresholds greater than 30 dBHL at frequencies of 3 and 4KHz. Such findings have already been demonstrated in other studies, since continuous exposure and high loudness tend to cause damage to high frequencies, reducing the amplitude of cochlear function^(23, 24)^.

According to the above, exposure to noise can, firstly, damage the outer hair cells, initially as a result of the mechanical rupture of the basilar membrane and the auditory sensory cells. These cells, as they are cochlear amplifiers, increase sound stimuli and, when reduced, compromise the functioning of the inner hair cells, which are true cochlear receptors and decoders. Thus, the audiological alterations found in musicians are anatomically and physiologically justified^(22)^.

In this sense, the findings demonstrate that measuring cochlear function, through electroacoustic procedures, such as otoacoustic emission, when no alterations are observed in the PTA, becomes important for adequate audiological monitoring and predicting future changes that may result from continuous and incorrect musical exposure.

However, despite this cochlear injury, musical exposure also tends to bring about benefits, mainly related to the central auditory nervous system. This finding is due to the fact that musical exposure can cause neuroplastic changes and compensations in the processing of the acoustic signal^(10,17)^. Thus, the need for continued health education on the risks of intensive sound exposure for musicians is highlighted, with an emphasis on the possible development of auditory and extra-auditory symptoms. The impacts and benefits of musical exposure demonstrate the need to emphasize effective hearing protection, that is, not only in the form of individual hearing protection, such as earplugs, but also in the form of noise-absorbing screens.

### Vestibular myogenic potentials in musicians

Musicians showed shorter latencies bilaterally for both components in cVEMP and for P15 in oVEMP. Similar findings have already been reported, of which when comparing the vestibular response with control groups, earlier response times were also observed, that is, better responses of the vestibulocollic and ocular reflexes were observed^(25)^. Thus, it is highlighted that musical stimulation contributes to the time and magnitude of response, both in the descending vestibular pathway (inferior vestibular nerve - cVEMP), and in the contralateral ascending superior vestibular pathway (superior vestibular nerve - oVEMP)^(11)^.

The act of playing an instrument or being exposed to musical practice becomes an extremely complex task. This is because several sensory systems are activated to perform a given task, needing to be coordinated with a high degree of synchrony and precision. Thus, during musical performance, sensory stimuli (auditory, visual and proprioceptive) and motor commands (articulatory, respiratory and limb coordination) are integrated^(25,26)^. Therefore, according to this complete multimodal stimulation, better vestibular responses are justified in this population. Thus, musicians appear to present better vestibular responses when compared to their control groups.

These findings allow us to infer that musicians are capable of performing compensatory movements with their heads, in response to oscillations or inclinations of the body during their musical performance (rehearsals and presentations), providing better functionality of the CVT. This finding is confirmed since, on both sides, the reflexes were triggered faster and with greater muscular intensity, justifying the results obtained in the present study.

Similarly, musicians, compared to non-musicians, are assumed to be able to make more precise ocular adjustments in order to maintain ocular fixation on the score or instrument while performing greater head movements. This condition provides a favorable performance for VOR, observed in the faster triggering bilaterally, and in a response of smaller muscular motricity on the right side and larger on the left side, although with a small discrepancy.

### Quality of life in musicians

The musicians in this study presented a considerably satisfactory QoL, with greater losses in the environmental domain. In contrast, a study^(27)^ that assessed the QoL of orchestra musicians found higher scores in the “environmental” and “vitality” domains, and lower scores in the “psychological” and “functional capacity” domains. This study indicated that such results are due to the high performance required in the population evaluated, demonstrating a significant impairment in QoL.

Research has found a significant relationship between emotional aspects and hearing loss. According to the authors, hearing complaints and, mainly, hearing loss, negatively influence the full use of musical skills and the perception of some tones and timbres, strongly interfering in the instrumentalist's QoL. However, it is necessary to consider personal ethical perspectives and characteristics, since QoL is intrinsic to an individual and influenced by the environment in which they are inserted, which causes variation from person to person and from one specific place to another^(27,28)^.

In the literature consulted, it was possible to observe that there are still few studies that seek to investigate the auditory, vestibular function and QoL in musicians, also directing a look at occupational performance, maximizing the accuracy of the results obtained in the VEMP of musicians and justifying the use of these potentials for monitoring the health and QoL of musicians in the work process.

Therefore, based on the results presented, the musicians in the present study may be exposed to lower sound intensity (amplifiers) when playing their instruments, and most of them practice individually or with few musicians. Furthermore, the average musical experience in years is reasonably low, the weekly sound exposure time is within what is considered adequate (around three hours per day), and the musicians presented hearing impairment in isolated frequencies (3 and 4kHz). Thus, it is believed that musicians have a more refined auditory perception as a result of musical practice and, consequently, faster vestibular reflexes, justified by the proximity of the structures (auditory and vestibular) and the anatomical and physiological functioning between them. Furthermore, studies have observed that during musical performance, complete multimodal stimulation (proprioceptive, auditory, motor and visual) occurs in an integrated manner, explaining such findings^(25,26)^.

The findings of the present study show that sound exposure may have influenced the positive results found in the present study, given that musical practice within tolerance limits, in terms of exposure time and sound intensity, is likely to provide auditory (auditory perception), vestibular and QoL benefits for musicians.

## CONCLUSION

When evaluating the influence of musical exposure on the hearing and vestibular reflexes of musicians, it was possible to observe higher thresholds at isolated frequencies (3 and 4KHz), faster triggering of vestibular reflexes than non-musicians, and a considerably satisfactory QoL.
