# Supplementary material for: Vestibulocochlear system and quality of life in musicians: a study on the effects of auditory exposure
Source: Codas. 2025 Feb 3;37(1):e20240010. doi: 10.1590/2317-1782/e20240010en (PMC11801196; doi:10.1590/2317-1782/e20240010en)
Supplement: Translation

# Translation: Sistema vestibulococlear e qualidade de vida de musicistas: um estudo acerca dos efeitos da exposição sonora

**DOI:** 10.1590/2317-1782/e20240010pt

**Authors:** Magali Scheuer, Bianca Nunes Pimentel, Priscilla dos Santos Martins, Hélinton Goulart Moreira, Valdete Alves Valentins dos Santos

**Affiliations:** 1 Programa de Pós-graduação em Distúrbios da Comunicação Humana, Universidade Federal de Santa Maria – UFSM - Santa Maria (RS), Brasil.; 2 Faculdade Unyleya - Rio de Janeiro (RJ), Brasil.; 3 Curso de Fonoaudiologia, Departamento de Fonoaudiologia, Universidade Federal de Santa Maria – UFSM - Santa Maria (RS), Brasil.

**Keywords:** Audição, Testes de Função Vestibular, Qualidade de Vida, Músico, Ruído

## Abstract

**Objetivo:**

Avaliar e comparar os efeitos da exposição sonora no sistema vestíbulococlear entre os grupos e a qualidade de vida dos musicistas.

**Método:**

Participaram 56 indivíduos, sendo 28 musicistas e 28 não-musicistas, com faixa etária de 18 a 45 anos e de ambos os sexos. Os participantes foram submetidos à anamnese, avaliação audiológica básica, potencial evocado miogênico vestibular (VEMP) e, exclusivamente, os musicistas ao questionário WHOQOL-Bref.

**Resultados:**

Participaram musicistas profissionais e entusiastas que apresentaram limiares auditivos aumentados nas frequências de 3 e 4KHz e qualidade de vida consideravelmente satisfatória. Houve significância nas latências do VEMP cervical e na latência da onda P15 do lado esquerdo do VEMP ocular do grupo estudo em relação ao grupo controle. O VEMP demonstrou ondas maiores bilateralmente nos homens em comparação com as mulheres.

**Conclusão:**

Concluiu-se que a exposição sonora, dentro dos limites de tolerância em tempo e intensidade, foi passível de proporcionar benefícios auditivos, vestibulares e de qualidade de vida para os musicistas.

## INTRODUÇÃO

A música e o ser humano estão intimamente ligados, isto é, a música sempre esteve presente na história da humanidade. Atualmente a música figura como ocupação profissional de milhões de pessoas em todo o mundo, atuando em vários âmbitos, e representando um importante papel sociocultural. Em razão disso, quando esses sujeitos são expostos, diversos benefícios podem ser constatados, principalmente relacionados aos mecanismos de facilitação da expressão e regulação emocional, o fortalecimento dos laços sociais, o sentimento de pertença, bem como o fomento da criatividade e do desenvolvimento cognitivo^(1).^

Sabe-se que a música exerce efeitos benéficos na qualidade de vida (QV) e que a prática musical sensibiliza a percepção auditiva através da seletividade perceptiva e fisiológica dos sons a nível coclear. Isso propicia a ativação córtico-coclear durante o treinamento musical, proporcionais ao tempo de exposição à prática^(2)^. Desse modo, a neuroplasticidade auditiva atua no aprimoramento do ajuste coclear e aumento da acuidade espectral, aprimorando a percepção auditiva.

Em contrapartida, os musicistas classificam-se como um grupo de risco para o desenvolvimento de alterações de origem ocupacional. Se tais profissionais forem expostos inadequadamente e por longos períodos a elevados níveis de pressão sonora, tal exposição pode desencadear danos auditivos e extra-auditivos (cefaleia, alterações no sistema imunológico, insônia, entre outros). Destacam-se, ainda, possíveis problemas musculoesqueléticos, quando relacionados à postura e esforço inadequados, tornando-se interessante mensurar o sistema vestíbulo-coclear e a QV nesta população^(3,4)^.

Para os musicistas é fundamental o conhecimento dos efeitos da exposição musical no funcionamento auditivo, mas ainda é incipiente no tocante à saúde vestibular (equilíbrio postural). Estudos recentes demonstram que a exposição sonora inadequada induz alterações auditivas e, também, acomete os órgãos vestibulares (sáculo e utrículo)^(5,6)^. Nesse sentido, os sintomas vestibulares podem anteceder perdas auditivas, ou seja, os possíveis déficits relacionados à saúde auditiva ocasionados pela prática profissional, além de problemas musculoesqueléticos e emocionais, juntos, podem interferir na sua carreira profissional e na qualidade de vida, tendo em vista a importância da percepção auditiva e corporal para essa categoria profissional.

Considerando a estreita relação anatomofisiológica entre os sistemas auditivo e vestibular, a exposição musical inadequada pode acometer tanto a cóclea quanto o sáculo e o utrículo, que são os principais responsáveis pela obtenção das variações de aceleração linear (horizontal e vertical) da cabeça e do corpo^(3)^. Diante disso, salienta-se a possibilidade de utilização do Potencial Evocado Miogênico Vestibular (VEMP), que propicia a compreensão dos reflexos do equilíbrio corporal (reflexo vestíbulo-ocular e reflexo vestíbulo-cervical), que atuam diretamente no equilíbrio corporal.

Visto que a pesquisa sobre a função auditiva e vestibular dos musicistas ainda é incipiente quando mensurada e relacionada à qualidade de vida, o presente estudo objetivou avaliar e comparar os efeitos da exposição sonora no sistema vestíbulococlear entre os grupos e a qualidade de vida dos musicistas.

## MÉTODO

O presente estudo foi aprovado pelo Comitê de Ética em Pesquisa, sob o parecer número 2.732.475 e CAAE 87348618.3.0000.5346. Trata-se de um estudo transversal, observacional, descritivo, que se utilizou de método quantitativo, desenvolvido no ambulatório de otoneurologia da instituição de origem, no período pré-pandêmico.

A amostra foi composta por conveniência, e todos os participantes realizaram os procedimentos individualmente, em dia e horário previamente agendados. Além disso, todos os participantes consentiram com a pesquisa, através da leitura e assinatura do Termo de Consentimento Livre e Esclarecido – TCLE. Os não-musicistas foram selecionados por voluntariado.

Adotaram-se como critérios de inclusão:

Grupo controle (GC): Idades entre 18 e 45 anos; limiares auditivos dentro dos padrões da normalidade; curvas timpanométricas indicando mobilidade normal do sistema tímpano-ossicular; presença em níveis normais dos reflexos acústicos contralaterais; apresentar condições mentais e cognitivas para responder e realizar a reprodução dos comandos solicitados; não apresentar queixa ou histórico de alterações neurológicas, traumáticas, cervicais ou oculares que impossibilitassem a realização dos procedimentos propostos, bem como doenças crônicas; não haver a exposição a riscos físicos, químicos e biológicos, de acordo com os limites de tolerância dispostos na Norma Regulamentadora 15^(7)^; evitar o uso de drogas vestíbulo tóxicas e relaxantes por 48 horas antes dos exames;
Grupo estudo (GE): Além dos critérios em comum entre os grupos (os mesmos listados para o GC), para este grupo foi inserido, tendo em vista o objetivo da pesquisa de evidenciar os achados audiológicos em musicistas: os indivíduos poderiam apresentar limiares auditivos iguais ou maiores a 30dBNA em qualquer frequência auditiva avaliada pela audiometria tonal liminar convencional (250 a 8000Hz), presença de queixas auditivas e vestibulares após o início da prática musical, devendo ser profissional da música (musicista), e possuir período de atuação superior a seis meses^(8)^.

Foram submetidos às avaliações 59 indivíduos, dos quais 56 atenderam aos critérios de inclusão, sendo estes divididos em dois grupos, pareados quanto ao sexo e idade:

Grupo Estudo (GE): Participaram 28 musicistas, sendo 21 (75%), do sexo masculino, e sete (25%), do sexo feminino, com média de idade de 25,07 anos;
Grupo Controle (GC): Composto por 28 indivíduos, que não fossem profissionais da música, ou que não tocassem nenhum tipo de instrumento musical.

Todos os sujeitos foram submetidos aos seguintes procedimentos:

**Anamnese previamente estruturada:** foi elaborada para a ocasião com questões relativas à história clínica, saúde auditiva e ocupacional, pregressa e atual. Além disso, os musicistas foram questionados quanto aos aspectos relacionados à prática musical;
**Inspeção do meato acústico externo:** realizada com o otoscópio MD 2.5V Omni 3000 LED Fibra Ótica, a fim de verificar possíveis obstruções que impossibilitassem a realização dos exames;
**Audiometria Tonal Liminar:** foi realizada por meio do audiômetro AD629 da marca Interacoustics com fone concha modelo TDH-39P. Avaliaram-se as frequências auditivas de 250 a 8000Hz por via aérea e, quando necessário, realizaram-se a pesquisa de via óssea, cujos resultados foram interpretados de acordo com os padrões para perda auditiva, quanto ao tipo e grau^(9)^;
**Logoaudiometria:** foi realizada a pesquisa do limiar de reconhecimento de fala, por meio de uma lista de palavras dissílabas apresentada em viva voz, considerando como resultado as intensidades (dB) em que os indivíduos atingiram 50% de acertos nas palavras apresentadas. Para avaliação do índice percentual de reconhecimento da fala, foi solicitada a repetição de uma lista de 25 palavras monossilábicas apresentada em viva voz, considerando-se os resultados com nenhuma dificuldade para compreender a fala aqueles que apresentaram de 100% a 92% de acertos^(9)^;
**Medidas de imitância acústica** (timpanometria e reflexos acústicos contralaterais): foram realizadas no equipamento AT235, da marca Interacoustics e fones tipo TDH-39. Para a classificação das curvas timpanométricas e para os reflexos acústicos utilizaram-se os critérios já propostos na literatura^(9)^;
**Potenciais Evocados Miogênicos Vestibulares:** Os Potenciais Evocados Miogênicos Vestibulares Cervical e Ocular foram realizados através do equipamento MASB ATC *Plus*, da marca *Contronic*. Primeiramente, foi realizada a limpeza da pele do participante com álcool absoluto, seguida de pasta abrasiva e fixação dos eletrodos com pasta eletrolítica. Destaca-se que para realização dos dois potenciais miogênicos vestibulares os indivíduos estavam sentados e que todos os participantes, de ambos os grupos, receberam orientações quanto à intensidade de apresentação dos estímulos sonoros do VEMP. Não foi apresentado nenhum esboço de expressão verbal e não-verbal de desconforto auditivo durante a avaliação.

Ambos os potenciais foram realizados com estímulo auditivo tone burst na intensidade de 118 dBNA e frequência de 500 Hz, apresentando no total de 200 estímulos, com a velocidade de apresentação de 5,1 estímulos por segundo, com filtro passa-banda de 10 a 1.500 Hz. O teste de impedância dos eletrodos foi inferior a 5 KΩ, e a diferença entre eles inferior a 2 KΩ^(10)^.

Foram apresentadas duas estimulações de cada lado, a fim de verificar a replicabilidade das respostas, com intervalos entre elas, permitindo o descanso da musculatura nos intervalos.

**Potencial Evocado Miogênico Vestibular Cervical (cVEMP):** o eletrodo ativo ipsilateral foi posicionado na borda anterior do músculo esternocleidomastoideo, em seu terço médio. O eletrodo de referência positivo ipsilateral foi posicionado acima da borda superior da clavícula, na linha esternal, e o terra, fixado na fronte (Fpz). Os participantes foram orientados a girar horizontalmente a cabeça para o lado oposto ao da orelha estimulada durante a estimulação sonora, contraindo o músculo esternocleidomastoideo. Foram marcadas as latências em milissegundos (ms) do primeiro pico positivo e do primeiro vale negativo, denominados P13 e N23^(10,11)^;
**Potencial Evocado Miogênico Vestibular Ocular (oVEMP):** o eletrodo ativo ipsilateral (negativo) foi colocado cerca de 1 cm abaixo da pálpebra inferior, no músculo oblíquo inferior. O eletrodo de referência contralateral (positivo) foi colocado a uma distância de 1 cm do eletrodo ativo, e o terra, fixado na fronte (Fpz). Os participantes foram orientados a manter a cabeça ereta e apenas desviar o olhar para cima, até o final da estimulação sonora. Foram marcadas as latências em milissegundos (ms) do primeiro vale negativo e do primeiro pico positivo, denominados N10 e P15, bilateralmente^(10,11)^.

Tanto para o cVEMP, como para o oVEMP, foram anotadas a latência, a amplitude dos picos e dos interpicos (interamplitude), em “microvolts” (uV). Os dados quantitativos (latências, amplitudes e interamplitudes) dos musicistas foram analisados com os valores de normalidade do equipamento do referido Laboratório e, também, foram comparados com os resultados do grupo controle. As análises e devidas marcações em todos os traçados foram submetidas à avaliação de duas juízas fonoaudiólogas, ambas especialistas na avaliação de pacientes com o protocolo do VEMP.

Para o GE, excepcionalmente, ainda foi aplicado o seguinte questionário, tendo em vista o objetivo do estudo de mensurar a qualidade de vida apenas dos musicistas após a comparação dos achados vestíbulo-cocleares entre os indivíduos musicistas e não musicistas.

**Instrumento de Avaliação de Qualidade de Vida - *The World Health Organization Quality of Life (WHOQOL- Bref)*:** trata-se de uma versão abreviada do Instrumento de Avaliação da Qualidade de Vida da Organização Mundial da Saúde^(12).^ Este instrumento é composto por 26 questões divididas em quatro domínios: físico, psicológico, social e ambiental e, ainda, duas perguntas gerais sobre a QV global. Os participantes responderam em uma escala de zero a cinco (quanto maior a pontuação melhor a QV), considerando as duas últimas semanas vividas. Os resultados foram somados segundo cada domínio, e os escores finais demonstraram a QV do indivíduo avaliado. Como não há um ponto de corte, quanto mais próximo de 100 melhor é a QV.

### Análise estatística dos dados

As variáveis qualitativas referentes aos dados de caracterização da amostra foram analisadas por estatística descritiva. Para a comparação dos valores do VEMP entre os grupos, primeiramente realizou-se o teste de *Shapiro-Wilk.* Este método foi empregado para testar a distribuição das variáveis quantitativas, a partir do qual foram selecionados: o Teste U de *Mann-Whitney*, para comparações entre duas amostras independentes; e o Teste de *Kruskal-Wallis* para comparações múltiplas, por meio do aplicativo computacional STATISTICA 9.1. O nível de significância considerado foi de 5% (α=0,05).

## RESULTADOS

### Caracterização da amostra para o GE

Para este grupo obteve-se média de 13,14 anos de experiência musical, de 14,96 horas semanais de exposição sonora (entre ensaios e apresentações) distribuídas em média de 5,39 dias na semana. Em relação à intensidade sonora a qual estão expostos conforme a sua própria percepção, constatando-se uma prevalência dos níveis elevados (57,14%) e de intensidade do ruído extra musical no momento de apresentação e ensaio predominantemente baixa (32,14%). Destaca-se que dentre as categorias de instrumentos, cinco músicos utilizam metal, cinco percussão, dois de madeira e 16 de corda.

Em relação aos aspectos e preferências musicais, foi observado que grande porcentagem dos participantes atua profissionalmente, com longo tempo de experiência e autopercepção elevada em relação à exposição sonora, distribuída em média durante cinco dias na semana (Tabela 1).

**Tabela 1:** Caracterização dos musicistas de acordo com os aspectos e preferências musicais (n=28)

**Variável**	**Categoria**	**Média (%)**
Instrumentista profissionalmente	Sim	60,71
	Não	39,29
Gêneros musicais	Popular	46,43
	Clássico	14,29
	Ambos	39,29
Intensidade (*Loudness*) da exposição sonora	Elevada	57,14
	Média	32,14
	Baixa	10,72
Intensidade (*Loudness*) do ruído extra musical	Elevada	21,43
	Média	28,57
	Baixa	32,14
	Inexistente	17,86
Preferência ao tocar	Acompanhado	82,14
	Solo	17,86
Posição em que toca	Sentado	42,86
	Em pé	17,86
	Ambos	39,29
Ambiente em que toca	Aberto	0,00
	Fechado	100,00
	Grande	42,86
	Pequeno	57,14

Legenda: n = número da amostra; % = porcentagem

Em relação à autopercepção dos sintomas auditivos e extra-auditivos após exposição sonora para o GE, evidenciou-se que, em média, 50% da amostra os percebeu. Desta amostra, foram observados como maior incidência: a dificuldade para compreender a fala em ambientes ruidosos, a intolerância aos sons, o estresse, a irritabilidade e prejuízos na concentração (Figura 1).

**Figura 1:** Caracterização dos musicistas de acordo com os sintomas auditivos e extra-auditivos após a exposição sonora (n=28)

Legenda: DAT = desconforto auditivo ao tocar; DVT = desconforto vestibular ao tocar; DCFR = dificuldade para compreender a fala em ambiente ruidoso; ISI = intolerâncias à sons intensos; MTL = mudança temporária de limiar; n = número da amostra

### Caracterização da acuidade auditiva periférica para o GE

Para a acuidade auditiva periférica, no GE, as médias dos limiares tonais aéreos apresentaram-se dentro dos padrões de normalidade, com simetria entre as orelhas. No entanto, 10,71% dos musicistas apresentaram limiar tonal igual ou maior a 30 dBNA nas frequências específicas de 3 e 4 kHz.

### Comparação dos resultados do cVEMP entre os grupos

Em relação às respostas miogênicas vestibulares cervicais, foram evidenciadas diferenças estatisticamente significantes para ambas as orelhas, relacionadas à latência dos componentes P13 e N23, com menores tempo de respostas para os musicistas (Tabela 2).

**Tabela 2:** Comparação entre os resultados obtidos no potencial evocado miogênico vestibular cervical (cVEMP), por orelha, dos grupos estudo e controle (n = 56)

**Lado**	**cVEMP**		**Estudo (n= 28)**		**Controle (n= 28)**		**Z**	**p-valor**
			**Média**	**DP**	**Média**	**DP**		
D	**P13**	**Latência**	17,51	1,21	18,99	2,24	-2,65	<0,01*
		**Amplitude**	59,36	44,40	43,45	22,53	0,98	0,32
	**N23**	**Latência**	26,65	2,43	27,82	2,42	-1,93	0,05*
		**Amplitude**	47,89	56,20	46,15	22,72	0,15	0,88
		**Interamplitude**	118,58	86,78	88,82	43,22	0,92	0,36
E*	**P13**	**Latência**	17,89	1,65	19,14	1,99	-2,60	<0,01*
		**Amplitude**	54,68	37,47	46,44	23,48	0,54	0,59
	**N23**	**Latência**	26,57	2,78	27,97	2,13	-2,28	0,02*
		**Amplitude**	43,21	59,46	55,22	27,17	-0,77	0,44
		**Interamplitude**	114,96	77,96	101,18	49,30	0,29	0,77

Teste *U de Mann-Whitney*; p < 0,05

* número da amostra igual à 27 indivíduos em ambos os grupos

Legenda: P = onda positiva; N = onda negativa; DP = desvio padrão; D = direito; E = esquerdo; n = número da amostra; Z = nível de confiança; p = valor de p

### Comparação dos resultados do oVEMP entre os grupos

Em relação às respostas miogênicas vestibulares oculares, foram evidenciadas diferenças significantes apenas para latência da orelha esquerda para o componente P15, com menor tempo de resposta para GE (Tabela 3).

**Tabela 3:** Comparação entre os resultados obtidos no potencial evocado miogênico vestibular ocular (oVEMP), por orelha, dos grupos estudo e controle (n = 56)

**Lado**	**oVEMP**		**Estudo (n= 28)**		**Controle (n= 28)**		**Z**	**p**
			**Média**	**DP**	**Média**	**DP**		
D	**N10**	**Latência**	13,02	1,31	13,73	1,34	-1,04	0,30
		**Amplitude**	2,65	3,62	3,50	4,13	-0,55	0,58
	**P15**	**Latência**	17,73	1,67	18,65	1,29	-1,53	0,12
		**Amplitude**	3,77	3,69	3,88	4,21	0,42	0,67
		**Interamplitude**	6,97	6,72	7,38	8,24	0,34	0,74
E*	**N10**	**Latência**	13,23	1,19	13,98	1,67	-1,49	0,14
		**Amplitude**	3,15	3,52	3,12	3,12	0,24	0,81
	**P15**	**Latência**	17,71	1,36	18,34	1,65	-2,32	0,02*
		**Amplitude**	4,53	4,23	4,29	5,09	0,88	0,38
		**Interamplitude**	8,09	7,21	7,98	9,53	0,75	0,45

Teste *U de Mann-Whitney*; p < 0,05

* número da amostra igual à 28 indivíduos em ambos os grupos

Legenda: P = onda positiva; N = onda negativa; DP = desvio padrão; D = direito; E = esquerdo; n = número da amostra; Z = nível de confiança; p = valor de p

### Representação gráfica dos potenciais evocados miogênicos

Na Figura 2 observa-se a representação gráfica do cVEMP, para ambas as orelhas, com menor latência para GE, e a representação gráfica do oVEMP, para ambas as orelhas, com menor latência para P15 para o GE.

**Figura 2:** Representação gráfica do cVEMP e oVEMP, por orelha

Legenda: cVEMP = reflexo vestíbulo cervical; oVEMP = reflexo vestíbulo ocular; GC = grupo controle; GE = grupo estudo; vermelho = orelha direita; azul = orelha esquerda; *diferença estatisticamente significante

### Caracterização da QV dos musicistas

No tocante à QV dos musicistas, a resposta foi consideravelmente satisfatória. Conforme as médias obtidas dos escores ao aplicar o questionário *WHOQOL-Bref*, o domínio ambiental (61.52) apresentou maior prejuízo, e o domínio físico (75.38) melhor escore. Entre estes, estão o domínio psicológico, com 72.17, e o domínio social, com 73.81, sendo 70.72 a média do escore geral de QV.

## DISCUSSÃO

A prática musical é relatada na literatura especializada como um importante instrumento de mudanças neuroplásticas no sistema vestíbulo-coclear^(13)^. Os achados apontam que a exposição correta pode proporcionar o aprimoramento da memória de trabalho e estimular as regiões corticais motoras e pré-motoras. Como consequência, torna-se importante aos sistemas auditivo e vestibular, em decorrência dos benefícios da plasticidade nestas regiões^(14,15)^.

A música proporciona mudanças na sensibilização da percepção auditiva, através da seletividade perceptiva e fisiológica dos sons a nível coclear, realizando uma ativação córtico-coclear e possibilitando maiores benefícios ao desempenho auditivo^(2,16)^. Desse modo, a neuroplasticidade auditiva atua no aperfeiçoamento do ajuste coclear e aumento da acuidade espectral, aprimorando a percepção auditiva e, consequentemente, contribuindo para uma melhor qualidade de vida^(17)^.

Tendo em vista tais manifestações, ressalta-se a importância da presente pesquisa, devido à carência de estudos que busquem mensurar tais aspectos relacionados ao equilíbrio corporal.

### Exposição musical e sintomas auditivos e extra-auditivos

Foi possível observar que os musicistas apresentaram queixas auditivas, principalmente, relacionadas à intolerância aos sons, desconforto vestibular e mudança transitória de limiar após prática musical. Ainda, evidenciaram-se efeitos extra-auditivos relacionados a dificuldades de concentração, irritabilidade e estresse. Tais achados corroboram com outras pesquisas que também observaram a presença dessas queixas^(18,19)^.

A presença de sintomas auditivos torna-se comum na população estudada, já que níveis de música e ruído altos podem danificar permanentemente o ouvido e, por um tempo, serem confortáveis e até mesmo imperceptíveis, dificultando medidas protetivas. Determinada pesquisa evidenciou que um grande número de adolescentes e jovens adultos americanos haviam experimentado mudanças temporárias de limiar e zumbido transitório após elevados níveis de exposição sonora^(20)^. Esses sintomas justificam-se pela hipótese de que a exposição a *loudness* elevada e contínua pode causar danos irreparáveis a alguma fração das células ciliadas e fibras nervosas da cóclea, mesmo quando os limiares audiométricos inicialmente se recuperam e os sintomas desaparecem^(21)^. Assim, o acúmulo de danos a exposições repetidas poderia causar problemas auditivos mais crônicos.

O surgimento e a possibilidade da cronicidade dos sintomas podem ocasionar diversos efeitos extra-auditivos, uma vez que, apesar de ser um ruído de lazer, a música ocasiona danos, mesmo que menores ― proteção do músculo do estapédio ―, mas semelhantes a outras atividades laborais^(22)^. Esses, podem ser justificados em decorrência da atividade laboral estar associada a atividades de estresse para um melhor desempenho, assim como decorrência dos impactos do ruído nas manifestações auditivas e corporais destes indivíduos, ocasionando a autopercepção dessas queixas.

Destaca-se que a incidência dos sintomas é proporcional ao instrumento utilizado, ou seja, uma pesquisa recente demonstra que o sintoma é diretamente proporcional à pressão sonora produzida pelo instrumento, bem como as características materiais e de posição^(18)^. Desse modo, justifica-se a presença das queixas auditivas e extra-auditivas.

### Exposição musical e acuidade auditiva periférica

Na presente pesquisa foi possível observar que os indivíduos músicos entusiastas ou profissionais, apresentaram limiares tonais aéreos maiores que 30 dBNA nas frequências de 3 e 4KHz. Tais achados já foram evidenciados em outros estudos, uma vez que a exposição contínua e a *loudness* elevada tende a causar danos nas frequências agudas, reduzindo a amplitude do funcionamento coclear^(23,24)^.

De acordo com o supracitado, a exposição ao ruído pode, primeiramente, lesionar as células ciliadas externas, inicialmente em decorrência da ruptura mecânica da membrana basilar e das células sensoriais auditivas. Estas células, por serem amplificadores cocleares, aumentam os estímulos sonoros e, quando reduzidas, comprometem o funcionamento das células ciliadas internas, que são verdadeiras receptoras e decodificadoras cocleares. Assim, justifica-se anatomofisiologicamente as alterações audiológicas encontradas em musicistas^(22)^.

Nesse sentido, os achados demonstram que mensurar o funcionamento coclear, por meio de procedimentos eletroacústicos, como a emissão otoacústica, quando não observada alterações na ATL, torna-se importante para um adequado monitoramento audiológico e preditivo de futuras alterações que possam ser decorrentes da exposição musical contínua e incorreta.

No entanto, apesar dessa lesão coclear, a exposição musical também tende a ocasionar benefícios, principalmente relacionados ao sistema nervoso auditivo central. Tal constatação se deve ao fato de que a exposição musical pode ocasionar mudanças neuroplásticas e compensações no processamento do sinal acústico^(10,17)^. Desse modo, salienta-se a necessidade da educação continuada em saúde sobre os riscos da exposição sonora intensiva aos músicos, com ênfase no possível desenvolvimento de sintomas auditivos e extra-auditivos. Os impactos e benefícios da exposição musical demonstram a necessidade de realçar uma efetiva proteção auditiva, ou seja, não apenas sob a forma de proteção auditiva individual, como tampões para os ouvidos, mas também em telas de absorção de ruído.

### Potenciais miogênicos vestibulares em musicistas

Os musicistas apresentaram latências menores bilateralmente para ambos os componentes no cVEMP e para P15 no oVEMP. Achados semelhantes já foram relatados, dos quais ao comparar a resposta vestibular com grupos controles, também foram observados tempo de resposta antecipados, ou seja, observa-se melhores respostas do reflexo vestíbulo-cólico e ocular^(25)^. Assim, salienta-se que a estimulação musical contribui para o tempo e magnitude de resposta, tanto na via vestibular descendente (nervo vestibular inferior- cVEMP), como na via vestibular superior contralateral ascendente (nervo vestibular superior- oVEMP)^(11)^.

O ato de tocar um instrumento ou estar exposto à prática musical torna-se uma tarefa extremamente complexa. Isso porque vários sistemas sensoriais são ativados para desempenhar determinada tarefa, necessitando serem coordenados com alto grau de sincronia e precisão. Desse modo, durante a execução musical, estímulos sensoriais (auditivos, visuais e proprioceptivos) e comandos motores (coordenação articulatória, respiratória e de membros) são integrados^(25,26)^. Portanto, de acordo com essa estimulação multimodal completa, justificam-se respostas vestibulares melhores nesta população. Assim, os musicistas parecem apresentar melhores respostas vestibulares quando comparados aos seus grupos controles.

Tais achados permitem inferir que os musicistas sejam capazes de realizar movimentos compensatórios com a cabeça, em resposta às oscilações ou inclinações do corpo durante sua performance musical (ensaios e apresentações), proporcionando melhor funcionalidade do RVC. Tal constatação ratifica-se uma vez que, em ambos os lados, os reflexos foram desencadeados mais rápido e em maior intensidade muscular, justificando os resultados obtidos no presente estudo.

Da mesma forma, presume-se que os musicistas, em comparação com os não-musicistas, sejam capazes de realizar ajustes oculares mais precisos, com o intuito de manter a fixação ocular na partitura ou no instrumento, enquanto realizam movimentos de cabeça com maior intensidade. Essa condição propicia um desempenho favorável ao RVO, constatado no desencadeamento mais rápido bilateralmente, e numa resposta de motricidade muscular menor no lado direito e maior no lado esquerdo, porém com pequena discrepância.

### Qualidade de vida em musicistas

Os musicistas da presente pesquisa apresentaram uma QV consideravelmente satisfatória, com maiores prejuízos no domínio ambiental. Em oposição, um estudo^(27)^ que avaliou a QV de musicistas de orquestras encontrou maiores escores nos domínios “ambiental” e “vitalidade”, e menores escores nos domínios “psicológico” e “capacidade funcional”. Este estudo apontou que tais resultados se devem ao alto rendimento exigido na população avaliada, demonstrando um prejuízo significativo na QV.

Pesquisas constataram uma relação significativa entre os aspectos emocionais e a perda auditiva. Segundo os autores, as queixas auditivas e, principalmente a perda auditiva, influenciam negativamente o uso pleno das habilidades musicais e percepção de alguns tons e timbres, interferindo fortemente na QV do instrumentista. Entretanto, é necessário considerar as perspectivas e características éticas pessoais, visto que a QV é intrínseca a um indivíduo e influenciada pelo meio onde está inserido, o que ocasiona uma variação de pessoa para pessoa e de um determinado local para outro^(27,28)^.

Na literatura consultada, pôde observar-se que ainda são escassos estudos que buscam investigar a função auditiva, vestibular e QV em musicistas, direcionando um olhar também para atuação ocupacional, maximizando a precisão dos resultados obtidos no VEMP dos musicistas e justificar a utilização desses potenciais para o acompanhamento da saúde e QV do músico no processo laboral.

Portanto, a partir dos resultados apresentados, os musicistas do presente estudo podem estar expostos a menor intensidade sonora (amplificadores) ao tocar seus instrumentos, e a maioria realiza a prática individualmente ou com poucos músicos. Ademais, a média de experiência musical em anos é razoavelmente baixa, o tempo de exposição sonora semanal está dentro do que se considera adequado (cerca de três horas por dia), e os musicistas apresentaram acometimento auditivo nas frequências isoladas (3 e 4kHz). Desse modo, acredita-se que os musicistas possuem uma percepção auditiva mais apurada em decorrência da prática musical e, consequentemente, reflexos vestibulares mais rápidos, justificados pela proximidade das estruturas (auditiva e vestibular) e funcionamento anatomofisiológico entre elas. Ainda, estudos observaram que durante a execução musical ocorre a estimulação multimodal completa (proprioceptiva, auditiva, motora e visual) de forma integrada, explicando tais achados^(25,26)^.

Os achados do presente estudo evidenciam que a exposição sonora pode ter influenciado nos resultados positivos encontrados no presente estudo, dado que a prática musical dentro dos limites de tolerância, em tempo de exposição e intensidade sonora, é passível de proporcionar benefícios auditivos (percepção auditiva), vestibulares e de QV para os musicistas.

## CONCLUSÃO

Ao avaliar a influência da exposição musical na audição e nos reflexos vestibulares de musicistas, foi possível constatar limiares maiores em frequências isoladas (3 e 4KHz), desencadeamento dos reflexos vestibulares mais rápidos do que não-musicistas, e uma QV consideravelmente satisfatória.
